# Supplementary material for: A synthetic BRET-based optogenetic device for pulsatile transgene expression enabling glucose homeostasis in mice
Source: Nat Commun. 2021 Jan 27;12:615. doi: 10.1038/s41467-021-20913-1 (PMC7840992; doi:10.1038/s41467-021-20913-1)

SUPPLEMENTARY INFORMATION

A synthetic BRET-based optogenetic device for pulsatile transgene expression enabling glucose homeostasis in mice

Supplementary Figures

Figure 1: BRET-based activation of LOVTRAP and CIBN-CRY2 systems by furimazine or blue light irradiance.

Figure 2: Schematic representation of the furimazine or blue light induced dimerization of luminGAVPO.

Figure 3: Furimazine- and light irradiance-dependent Gluc expression.

Figure 4: The effects of Nluc-GAVPO content on the furimazine-induced Gluc expression.

Figure 5: Furimazine-dependent luminescence intensities.

Figure 6: Fluorescence imaging of furimazine-induced BFP mRNA expression.

Figure 7: Negligible cytotoxicity of furimazine.

Figure 8: Luminescence profiles for the cells induced by different frequencies of furimazine.

Figure 9: RT-qPCR analysis of furimazine-induced Gluc mRNA transcription.

Figure 10: Pulsatile activation of gene expression by luminGAVPO.

Figure 11: Construction of the cell lines stably expressing Gluc and insulin under the control of LuminON system.

Figure 12: Light-induced glycemic control in T1D mice.

Figure 13: Antidiabetic efficacy of LuminON system-controlled insulin expression in T1D mice during feeding.

Figure 14: Toxicity of furimazine in vivo.

Figure 15: Maximal tolerated dosage of furimazine by oral administration.

Supplementary Tables

Table 1: P values in the figures.

Table 2: Primers for RT-qPCR.

Supplementary Notes

Note 1: Sequence information.

Note 2: The original WB figures of luminGAVPO.

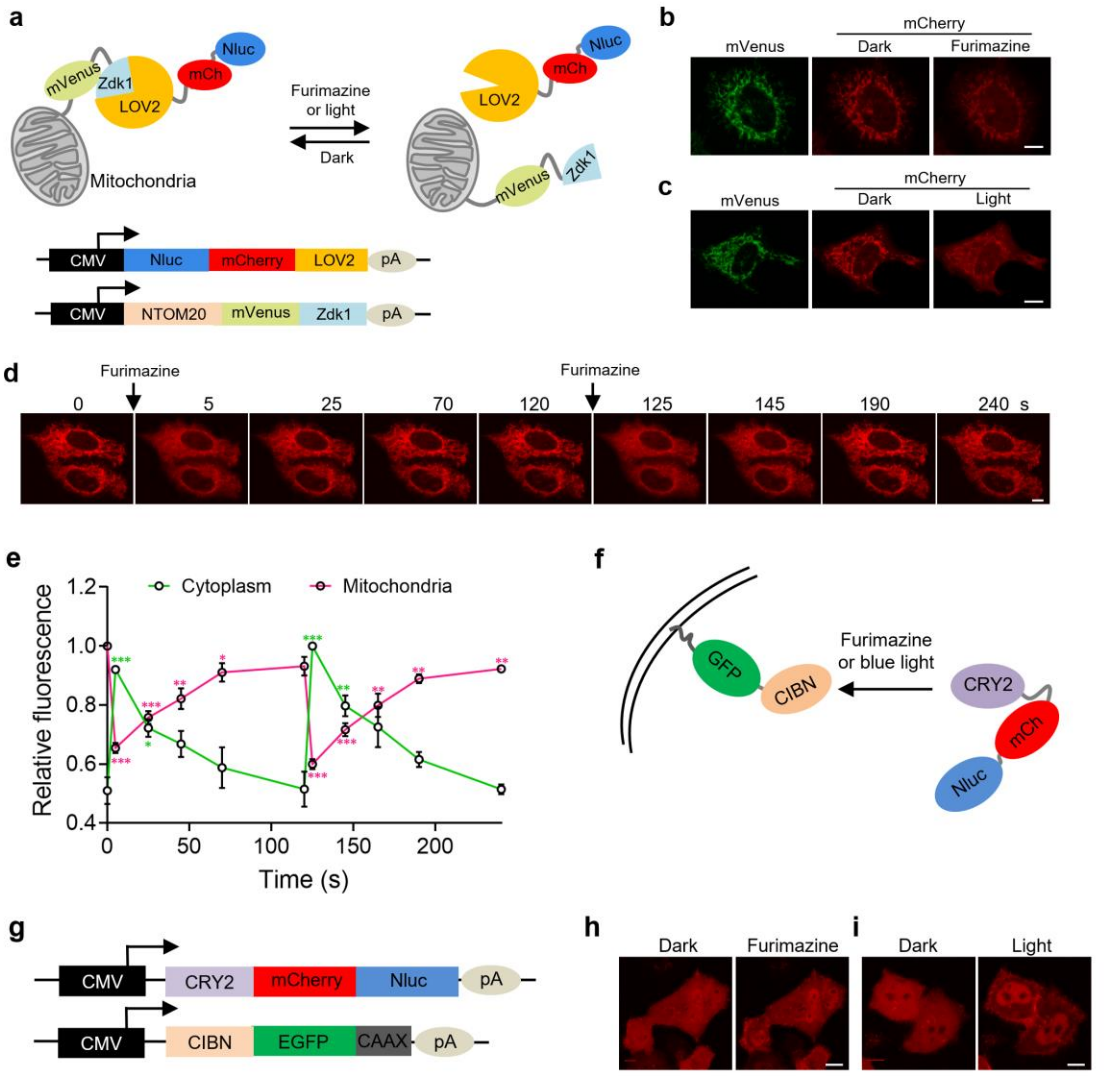

Supplementary Fig. 1 BRET-based activation of LOVTRAP system or CIBN-CRY2 system by furimazine or light irradiance. (a) Schematic showing BRET-based activation of LOVTRAP system by Nluc. **(b-c)** Fluorescence imaging of HeLa cells expressing Nluc-mCherry-LOV2 and NTOM20-mVenus-Zdk1 before and after induction by 20 µM furimazine **(b)** or 25 µW blue light irradiance **(c)**. Scale bar, 10 µm. **(d)** Imaging and **(e)** quantification of furimazine-induced translocation of Nluc-mCherry-LOV2 from the outer membrane of mitochondria to the cytoplasm and return to the outer membrane of mitochondria as light dims to the dark state. Cells were treated with 20 µM furimazine spaced 120 s apart during two consecutive furimazine-dark cycles. Scale bar, 10 µm. Statistical comparison was performed by a two-tailed *t* test. *$P < 0.05$, **$P < 0.01$, ***$P < 0.001$, versus control. Data represent the mean ± s.d. (n = 5 cells). **(f)** Schematic showing BRET-based activation of CIBN-CRY2 system by Nluc. **(g-i)** Nluc was fused to the C terminus of CRY2-mCherry fusion protein. When it was co-expressed with

the CIBN-pmEGFP with a CAAX box prenylation motif allowing for targeting to the plasma membrane, the mCherry fluorescence accumulated at plasma membrane after induction by 20 μM furimazine or exposure to 25 μW blue light. Scale bar, 10 μm. For **b, c, d, h** and **i**, at least two independent experiments were carried out with similar results. Source data are provided as a Source data file.

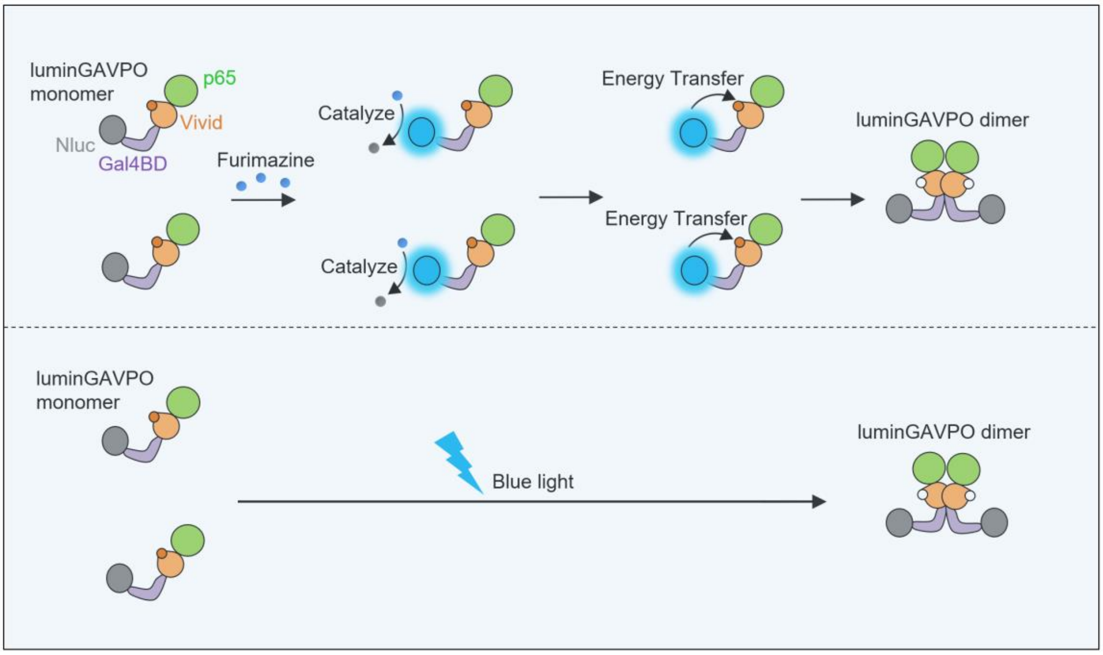

Supplementary Fig. 2 Schematic representation of the furimazine or blue light induced dimerization of luminGAVPO. In the presence of furimazine, Nluc domain in luminGAVPO catalyzes furimazine to produce blue light (spectral maximum 454 nm), which in turn activates the Vivid LOV domain by bioluminescence resonance energy transfer (BRET), resulting in dimerization of luminGAVPO. Blue light can directly activate Vivid domain to induce dimerization of luminGAVPO.

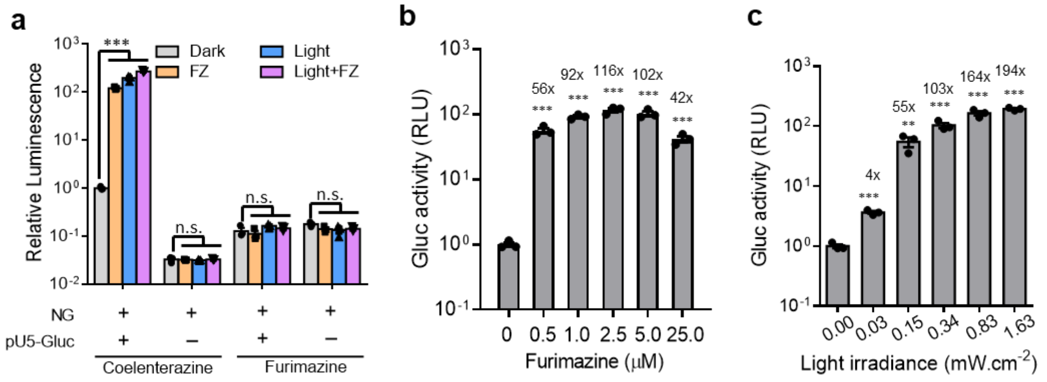

Supplementary Fig. 3 Furimazine- and light irradiance-dependent Gluc expression. (a) HEK293 cells transfected with NG and pU5-Gluc plasmids or NG plasmid alone were incubated with furimazine and/or exposed to blue light for 24 h and luminescence profiles were recorded by coelenterazine or furimazine. The data were normalized to the cells transfected with NG and pU5-Gluc kept under dark conditions without any other treatment. Data represent the mean ± s.d. from three technical replicates. **(b, c)** HEK293 cells cotransfected with luminGAVPO and pU5-Gluc were induced by various concentrations (0–25 µM) of furimazine **(b)** or different blue light intensities (0-1.6 mW/cm^2) **(c).** Gluc activities were determined 24 h after induction. The data were normalized to the cells kept in dark without any other treatment. Data in **(a-c)** represent the mean ± s.d. from three technical replicates. Statistical comparison was performed by a two-tailed *t* test. n.s. means not significantly different, $^{**}P < 0.01$, $^{***}P < 0.001$, versus control. Source data are provided as a Source data file.

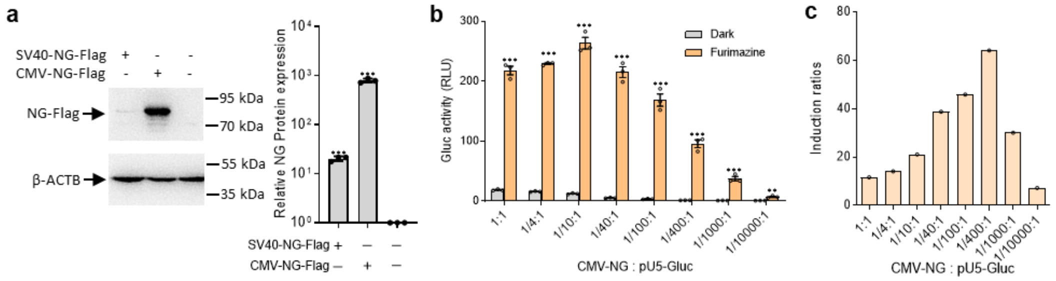

Supplementary Fig. 4 The effects of Nluc-GAVPO content on the furimazine-induced Gluc expression. (a) Western blot analysis of Nluc-GAVPO expression driven by SV40 or CMV promoter. Cells transfected with empty plasmid were used as negative control. **(b-c)** Optimization of the LuminON system by adjusting different plasmid ratios of the activator CMV-NG and the reporter pU5-Gluc. HEK293 cells co-transfected with a constant amount of the reporter pU5-Gluc and various amounts of CMV-NG were cultured in dark in the presence or absence of furimazine. **(b)** Gluc expression were determined 24 h after transfection and **(c)** the induction ratios were calculated accordingly. The data were normalized to the cells kept in dark without any other treatment. Statistical comparison was performed by a two-tailed *t* test. $^{**}P < 0.01$, $^{***}P < 0.001$, versus control. The data are shown as the mean ± s.d. from three technical replicates. Source data are provided as a Source data file.

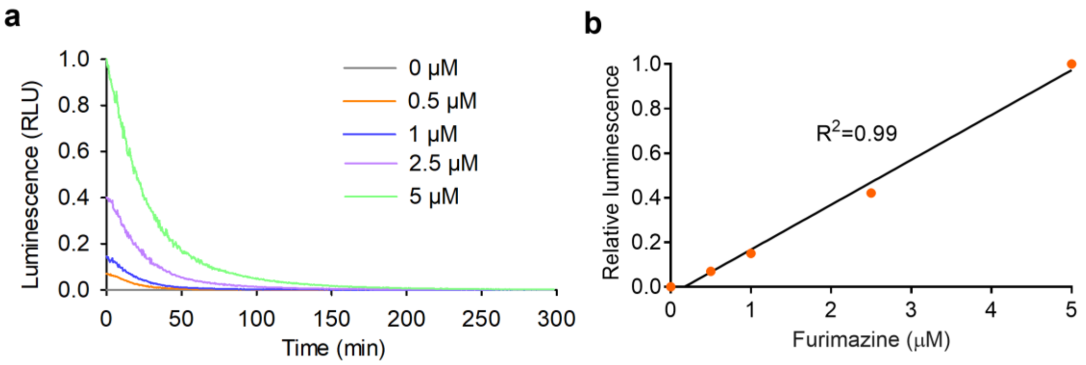

Supplementary Fig. 5 Furimazine-dependent luminescence intensities. (a) Characterization of the luminescence intensities of HEK293 cells equipped with luminGAVPO and incubated with different concentrations of furimazine. **(b)** Fitting curve of the maximal luminescence intensities of different furimazine concentrations in **(a)**. Source data are provided as a Source data file.

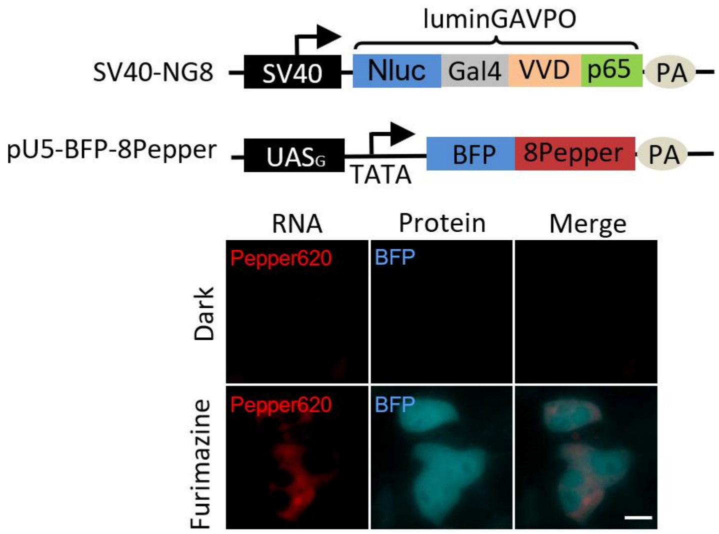

Supplementary Fig. 6 Fluorescence imaging of furimazine-induced BFP mRNA expression. HEK293 cells expressing BFP-8Pepper RNA under the control of the LuminON system were induced by 2.5 μM furimazine. Imaging of Pepper620 and BFP fluorescence was performed after the cells incubated with 1 μM HBC620. Scale bars, 10 μm. At least two independent experiments were carried out with similar results.

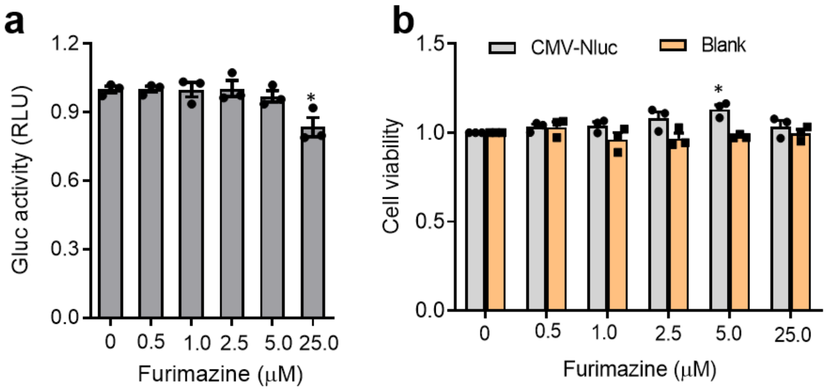

Supplementary Fig. 7 Cytotoxicity assay of furimazine in human cells. HEK293 cells expressing Gluc **(a)** or Nluc **(b)** driven by a CMV constitutive promoter were cultured upon incubation with different concentrations of furimazine (0-25 μM) for 48 h. Gluc activities were determined **(a)** and cell viability was measured by the CCK-8 assay **(b)**. In **(b)**, cells transfected with empty plasmid expressing no Nluc were used as the controls. Data were normalized to the cells kept in dark without any other treatment. Statistical comparison was performed by a two-tailed *t* test. **P* < 0. 05. Data represent the mean ± s.d. from three cell cultures. Source data are provided as a Source data file.

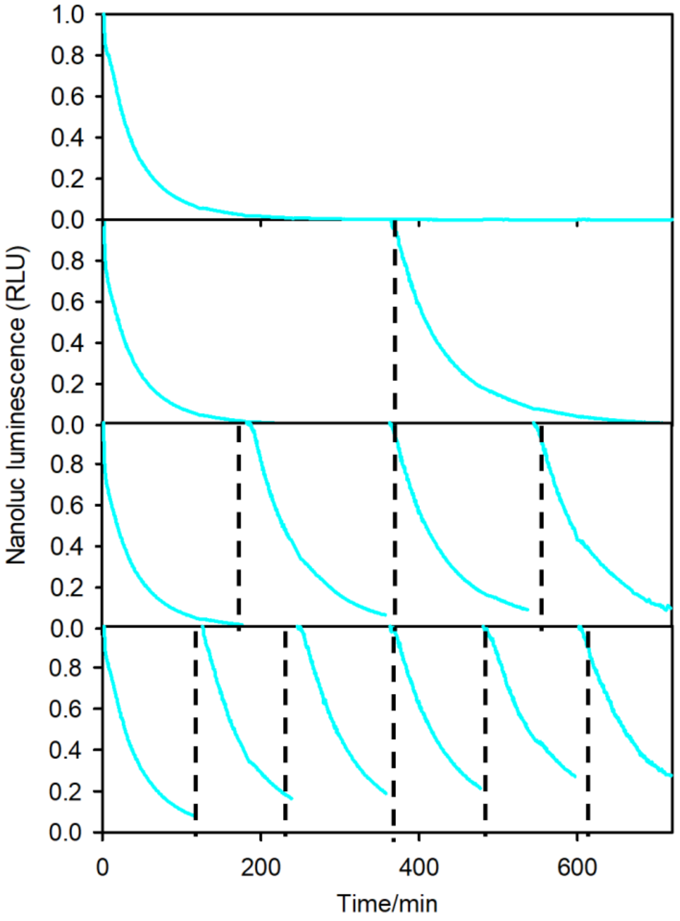

Supplementary Fig. 8 Luminescence profiles of the cells induced by different frequencies of furimazine. HEK293 cells transfected with luminGAVPO and pU5-Gluc were induced by different frequencies of 2.5 μM furimazine within 12 h, and luminescence profiles were recorded. Source data are provided as a Source data file.

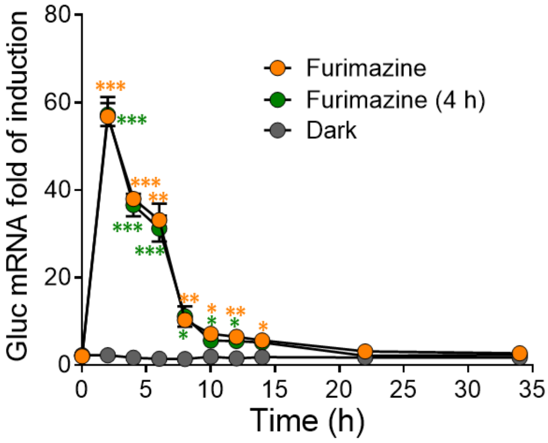

Supplementary Fig. 9 RT-qPCR analysis of furimazine-induced Gluc mRNA transcription. LuminON system-mediated Gluc mRNA transcription in HEK293 cells incubated with 2.5 μM furimazine for 4 h or 35 h. Gluc mRNA levels at the indicated time points were analyzed using RT-qPCR. Data were normalized to the sample at time 0. Statistical comparison was performed by a two-tailed *t* test. **P* < 0.05, ***P* < 0.01, ****P* < 0.001 versus control. Data represent the mean ± s.d. from three technical replicates. Source data are provided as a Source data file.

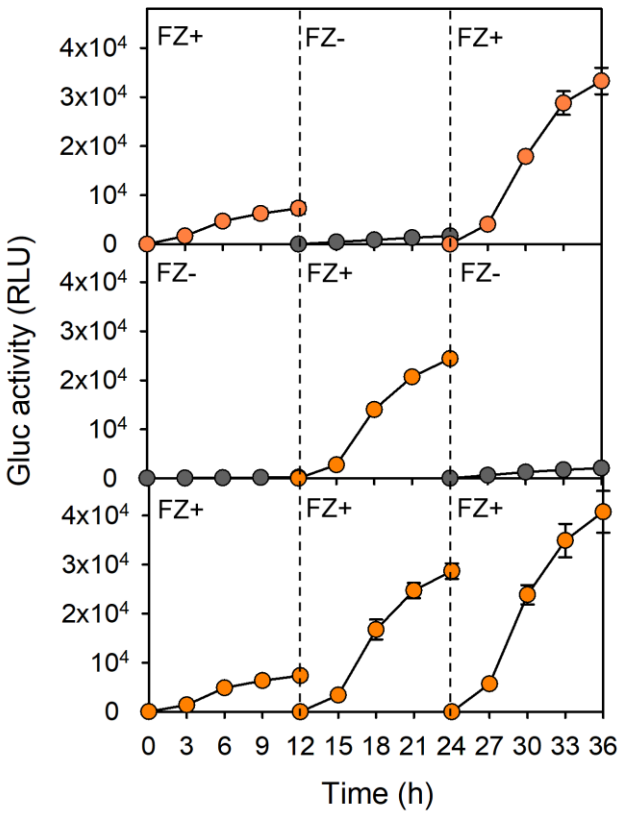

Supplementary Fig. 10 Reversibility of Gluc expression mediated by luminGAVPO. HEK293T cells engineered with the luminGAVPO system were cultivated for 36 h while alternating the furimazine concentrations (25 μM, ON; 0 μM, OFF) every 12 h. Gluc expression was profiled every 3 h. Data represent the mean ± s.d. from three technical replicates. Source data are provided as a Source data file.

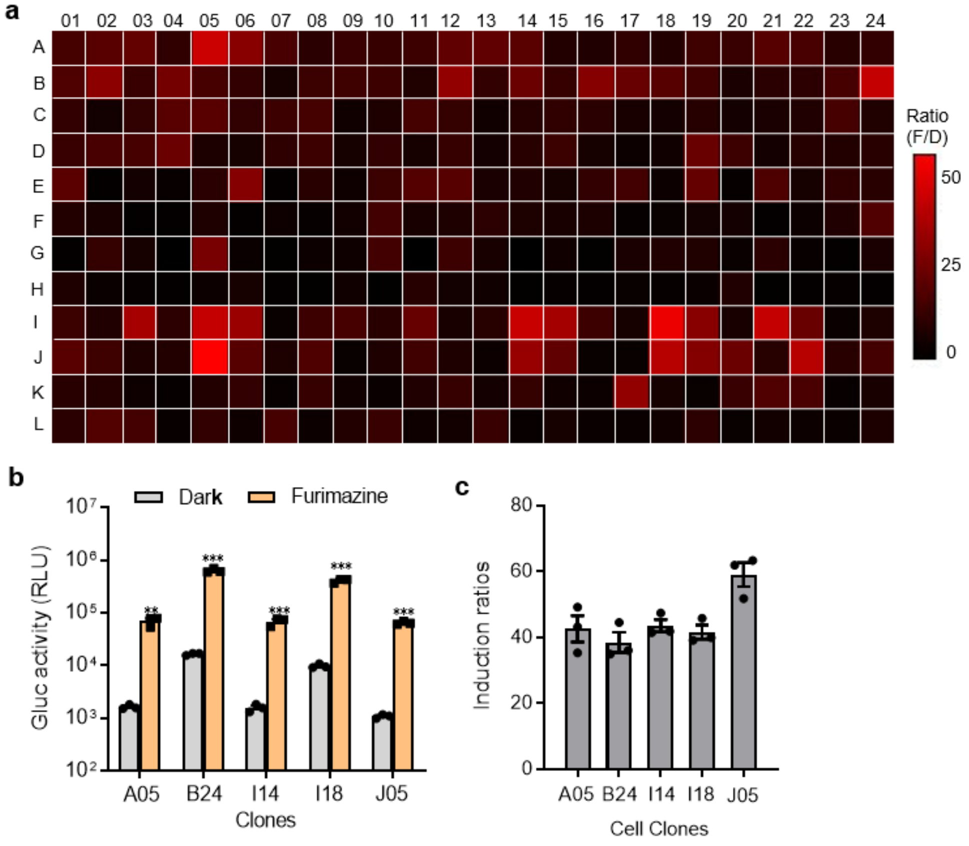

Supplementary Fig. 11 Construction of the HEK293$_{Gluc\text{-}P2A\text{-}mINS}$ stable cell lines stably expressing Gluc and insulin under the control of LuminON system. (a) HEK293T cells were cotransfected with pYH88 (ITR-P$_{CMV}$-NG: P$_{mPGK}$-PurR-pA-ITR) and pWS251 (ITR-P$_{U5}$-Gluc-P2A-mINSpA:P$_{mPGK}$-ZeoR-P2A-EGFP-pA-ITR) and cultured upon incubation with puromycin and zeocin for 2 weeks. 288 clones were randomly picked and cultured in the presence or absence of furimazine. Gluc expression was determined 24 h after induction and the induction ratios were calculated. Every rectangular grid corresponding to 1-24 and A-L in the figure represents different cell clones. **(b-c)** Several clones with high induction performance in **(a)** were further characterized for their furimazine-induced Gluc expression **(b)** and induction ratios **(c)**. Statistical comparison was performed by a two-tailed *t* test. ***P* < 0.01, ****P* < 0.001 versus control. Data in **(b)** and **(c)** represent the mean ± s.d. from three technical replicates. Source data are provided as a Source data file.

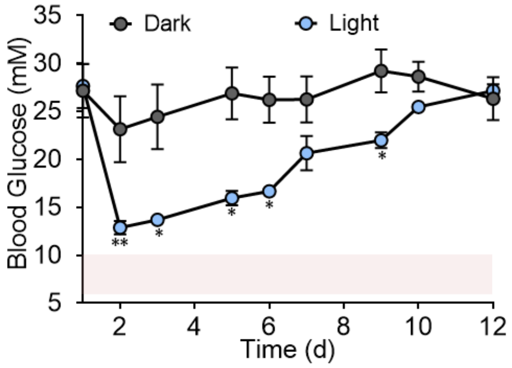

Supplementary Fig. 12 Light-induced glycemic control in T1D mice. The implanted T1D mice were illuminated with 90 mW·cm^{-2} blue light for 6 h before glucose levels were determined every day. Statistical comparison was performed by a two-tailed *t* test. *$P < 0.05$, **$P < 0.01$ versus control. Pink area represents normal blood glucose range. Data represent the mean ± s.d. from 5 mice. Source data are provided as a Source data file.

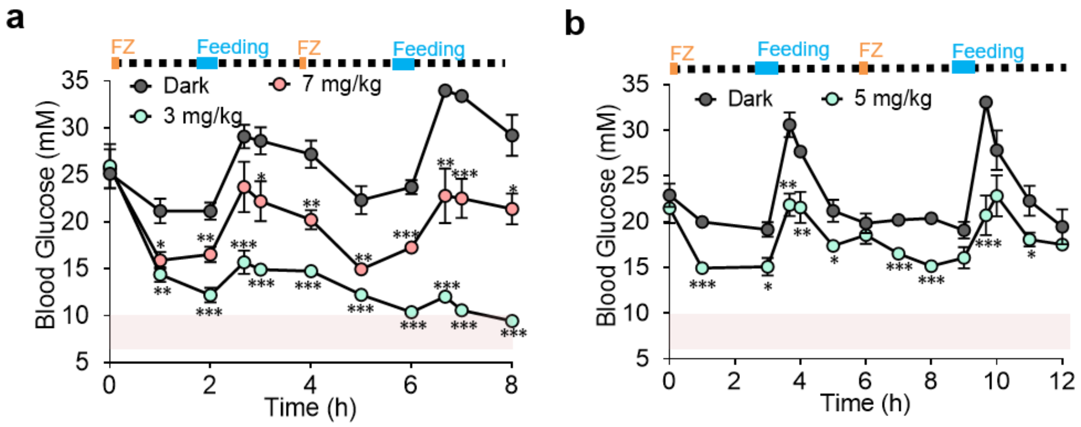

Supplementary Fig. 13 Antidiabetic efficacy of LuminON system-controlled insulin expression in T1D mice during feeding. The implanted mice were administered with 3, 5 or 7 mg/kg of furimazine by IG at **(a)** 2 h or **(b)** 3 h before feeding, and blood glucose levels were monitored. All the statistical comparison was performed by two-tailed *t* test. **P* < 0.05, ***P* < 0.01, ****P* < 0.001 versus control. Pink area represents normal blood glucose range. Data represent the mean ± s.d. from 5 mice. Source data are provided as a Source data file.

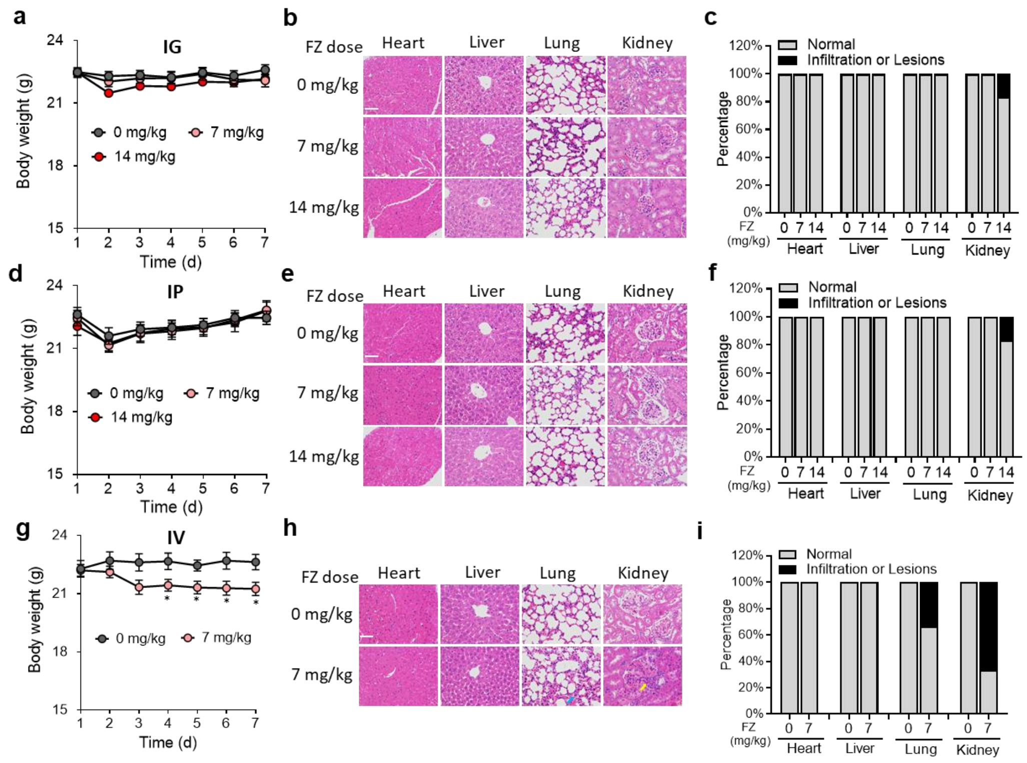

Supplementary Fig. 14 Toxicity of furimazine in vivo. (a-i) Mice were administrated with different dosages of furimazine (FZ) twice daily in the range of 0-14 mg/kg, via intragastric (IG) **(a-c)**, intraperitoneal (IP) **(d-f)** or intravenous (IV) **(g-i)** administration. **(a, d, g)** Body weight was monitored every day for a total of 7 days. Data represent the mean ± s.d. from 6 mice. Statistical comparison was performed by two-tailed *t* test. n.s. means not significantly different, **P* < 0.05, ***P* < 0.01 versus control. **(b, e, h)** Hematoxylin and eosin (H&E) staining for the indicated tissues in animals treated with different amounts of furimazine (FZ) via IG **(b)**, IP **(e)** or IV **(h)** administration. Blue and yellow arrows in **(f)** indicate alveolar stenosis in lung and granulocyte infiltration in kidney, respectively. H&E staining, × 400 magnification. Scale bars, 50 µm. **(c, f, i)** Quantification of the sections in **(b)**, **(e)** and **(h)** with cell infiltration or lesions. The data represent the percentage of the sections with and without cell infiltration or lesions from six sections. For **b**, **e** and **h**, at least two independent experiments were carried out with similar results. Source data are provided as a Source data file.

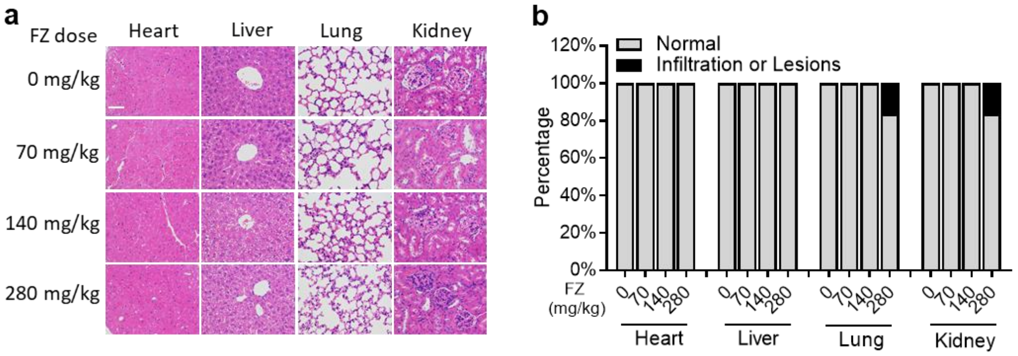

Supplementary Fig. 15 Maximal tolerated dosage of furimazine by oral administration. (a) Hematoxylin and eosin (H&E) staining for the indicated tissues in animals treated with single dose of different amounts of furimazine (FZ) in the range of 0-280 mg/kg via intragastric (IG) administration. H&E staining, × 400 magnification. Scale bars, 50 µm. **(b)** Quantification of the sections in **(a)** with cell infiltration or lesions. The data represent the percentage of the sections with and without cell infiltration or lesions from six sections. For **a**, at least two independent experiments were carried out with similar results. Source data are provided as a Source data file.

Supplementary Table 1 *P* values in the figures

Fig.1c			
Dark VS.	FZ	Light	Light+FZ
	P value	*P* value	*P* value
Nluc+GAVPO	0.2789	7.3E-06	0.000286
CMV-NG	0.00015	0.00365	0.000511
CMV-VN	0.00144	0.01969	0.0007
CMV-PN	0.01997	0.03304	0.005963
SV40-NG	2.2E-05	6.6E-07	0.000129
SV40-VN	3.8E-05	0.00059	0.000787
SV40-PN	0.00064	0.0007	0.000106

Fig.1f			
Dark VS.	FZ	Light	Light+FZ
	P value	*P* value	*P* value
PC-3	1.1E-05	7.6195E-05	2.482E-05
A549	0.00492	2.3617E-06	3.55E-05
H1299	6.1E-05	3.0438E-05	8.497E-05
COS7	0.00013	1.4543E-05	2.054E-06
U87	2.4E-05	0.00042271	1.384E-05
293T	9.4E-07	0.000271	2.727E-05
HELA	0.00151	1.5916E-05	4.467E-05

Fig.3e
Dark VS. FZ
P value
2.0694E-07

Fig.3g	
Dark VS. FZ	
Time (h)	*P* value
0	0.72921
2	3.5E-06
4	2.1E-05
6	4.1E-05
8	2.7E-06
10	2.2E-05
12	0.00014
14	7.9E-07
16	2.9E-05

Fig.1e			
Dark VS.			
1×	2×	4×	6×
P value	*P* value	*P* value	*P* value
0.0004	1.6E-07	2.9E-06	0.000143

Fig.3f				
Dark VS.	FZ-2.5mg/kg	FZ-3mg/kg	FZ-4mg/kg	FZ-5mg/kg
Time (h)	*P* value	*P* value	*P* value	*P* value
0	0.97958	0.98609338	0.9539826	0.9434898
2	0.00296	3.9271E-05	0.0003538	0.0056062
4	0.0023	0.00043524	0.0043695	5.761E-05
6	0.00412	0.00037947	0.0005312	0.0008108
8	0.06887	0.00054391	0.0099305	4.216E-05

Supplementary Table 1 P values in the figures

Fig.4b			
Implant + FZ VS.	None	FZ	Implant
	P value	P value	P value
	0.00032907	4.98802E-05	0.00038004

Fig.4c			
Dark VS.	FZ-IG	FZ-IP	FZ-IOCV
Time (h)	P value	P value	P value
0	0.7429673	0.987299566	0.7855933
1	0.00015308	0.014102809	0.00030389
2	0.00037828	0.006850983	8.558E-05
4	0.000512	0.002948579	0.00034379
6	0.01225589	0.000172437	0.00172128

Fig.4d			
Dark VS.	FZ-3mg/kg	FZ-5mg/kg	FZ-7mg/kg
Time (h)	P value	P value	P value
0	0.71198806	0.939229631	0.37388796
1	0.00035347	0.000565424	0.00054955
2	0.00262543	0.000855653	0.00045014
3	0.02517029	0.002308741	0.00016043
4	0.02103093	0.011634584	0.00017955
6	0.57637734	0.292487613	0.01038304

Fig.4e	
Dark VS. FZ	
Time (min)	P value
0	0.207537
15	0.1742385
30	0.0244814
60	0.0009961
90	0.0045656
120	0.019693

Fig.4g	
Dark VS. FZ	
Time (days)	P value
1	0.9771996
2	0.0095914
3	0.0076759
5	0.0010667
6	0.0009148
7	0.0009991
9	0.0002738
10	4.391E-05
12	0.0015481
13	5.305E-05
14	0.0313311

Fig.4f	
Dark VS. FZ	
Time (days)	P value
1	0.74097324
2	0.00038881
5	0.00060703
7	0.00012454
11	9.259E-05

Fig.4i	
Dark VS. FZ-5mg/kg	
Time (h)	P value
0	0.50624875
1	0.00233475
2	0.00027547
2.67	9.0483E-05
3	0.00073511
4	0.00373771
5	0.00124897
6	8.3057E-05
6.67	7.7428E-08
7	3.3393E-07
8	0.0005123

Fig.4j	
Dark VS. FZ-7mg/kg	
Time (h)	P value
0	0.9606185
1	0.001602
3	0.0002138
3.67	1.605E-05
4	4.401E-05
5	0.0007552
6	0.0118216
7	8.366E-06
8	0.0001923
9	0.0021
9.67	1.362E-06
10	0.0033886
11	0.0025793
12	0.0108721

Supplementary Table 1 P values in the figures continued

Supplementary Fig. 1e		
0 s VS.	Cytoplasm	Mitochondria
Time (s)	*P* value	*P* value
5	0.000834	3.49238E-05
25	0.017537	0.000352144
45	0.067791	0.007071895
70	0.39884	0.04449228
120	0.949538	0.099220093
125	0.000419	2.00836E-05
145	0.007391	0.000229023
165	0.058091	0.007013742
190	0.112439	0.001586259
240	0.925475	0.002096564

Supplementary Fig. 3a				
	Dark VS.	FZ	Light	Light+FZ
		P value	*P* value	*P* value
Coelenterazine	NG+pU5-Gluc	1.3E-05	0.00017113	4.91E-06
	NG	0.84995	0.7530737	1
Furimazine	NG+pU5-Gluc	0.55143	0.16978029	0.484492
	NG	0.08268	0.13593298	0.07313

Supplementary Fig. 4a		
Blank VS.	SV40-NG-Flag	CMV-NG-Flag
	P value	*P* value
	0.000117347	5.96734E-05

Supplementary Fig. 4b	
Dark VS.	Furimazine
CMV-NG : pU5-Gluc	*P* value
1:1	1.01E-05
1/4:1	1.41E-08
1/10:1	1.375E-05
1/40:1	2.078E-05
1/100:1	8.933E-05
1/400:1	0.0001273
1/1000:1	0.0006078
1/10000:1	0.003817

Supplementary Fig. 7b		
FZ-0μM VS.	CMV-Nluc	Blank
FZ (μM)	*P* value	*P* value
0.5	0.170102	0.387969669
1	0.195297	0.437374189
2.5	0.158446	0.40070885
5	0.032554	0.08373961
25	0.394884	0.875638506

Supplementary Fig. 3b	
FZ-0μM VS.	FZ
FZ (μM)	*P* value
0.5	0.000428657
1	2.44882E-05
2.5	0.000137523
5	0.000280977
25	0.000726339

Supplementary Fig. 3c	
Dark VS.	Light
Irradiance (mW/cm2)	*P* value
0.03	0.000224
0.15	0.00554
0.34	0.000571
0.83	0.000259
1.63	4.75E-06

Supplementary Fig. 7a	
FZ-0μM VS.	FZ
FZ (μM)	*P* value
0.5	0.9524304
1	0.9829104
2.5	0.9216826
5	0.3660277
25	0.0210559

Supplementary Table 1 *P* values in the figures continued

Supplementary Fig. 9		
Dark VS.	FZ 4h	FZ
Time (h)	*P* value	*P* value
0	0.8836	0.64899
2	4.04E-05	0.00029
4	0.000179	7.6E-06
6	0.000581	0.00112
8	0.014458	0.00192
10	0.029267	0.02117
12	0.037146	0.00994
14	0.050975	0.01439
22	0.71953	0.15971
34	0.166202	0.08139

Supplementary Fig. 11b	
Dark VS.	FZ
Cell clones	*P* value
A05	0.00109063
B24	3.7384E-05
I14	0.00070327
I18	4.9221E-05
J05	1.2772E-05

Supplementary Fig. 12	
Dark VS.	Light
Time (days)	*P* value
1	0.89350845
2	0.00925016
3	0.01370634
5	0.01037944
6	0.01050456
7	0.11710528
9	0.02847908
10	0.12519812
12	0.75892495

Supplementary Fig. 13a		
Dark VS.	FZ-3mg/kg	FZ-7mg/kg
Time (h)	*P* value	*P* value
0	0.8149473	0.7509159
1	0.0104621	0.00223351
2	0.0058215	7.7137E-05
2.67	0.1067636	6.3142E-05
3	0.0360213	2.3104E-05
4	0.0043794	3.6374E-05
5	0.0019534	0.00015731
6	0.0001701	1.432E-07
6.67	0.0048218	6.2021E-10
7	0.0009916	3.5299E-09
8	0.0206237	2.2684E-05

Supplementary Fig. 13b	
Dark VS. FZ-5mg/kg	
Time (h)	*P* value
0	0.5135
1	0.0002
3	0.0119
3.67	0.0013
4	0.0094
5	0.0210
6	0.3955
7	0.0004
8	0.0000
9	0.0750
9.67	0.0005
10	0.1483
11	0.0476
12	0.3629

Supplementary Table 1 *P* values in the figures continued

Supplementary Fig. 14a		
0mg/kg VS.	FZ-7mg/kg	FZ-14mg/kg
Time (d)	*P* value	*P* value
1	0.8703653	0.9593124
2	0.4381677	0.0065258
3	0.6199245	0.0435301
4	0.9268748	0.2089537
5	0.8447448	0.1238339
6	0.6188881	0.3483665
7	0.2122318	0.1659813

Supplementary Fig. 14d		
0mg/kg VS.	FZ-7mg/kg	FZ-14mg/kg
Time (d)	*P* value	*P* value
1	0.73039368	0.3245248
2	0.37488469	0.5533728
3	0.70357006	0.7212501
4	0.74073662	0.9062734
5	0.74305901	0.8350292
6	0.83617852	0.7135568
7	0.48365481	0.5894169

Supplementary Fig. 14g	
0mg/kg VS.	FZ-7mg/kg
Time (d)	*P* value
1	0.87644459
2	0.30488068
3	0.05028049
4	0.0414655
5	0.02590412
6	0.03016127
7	0.02590892

Supplementary Table 2 Primers for RT-qPCR

Primer name	Sequence
**Gluc forward**	GCCAATGCCCGGAAAGCT
**Gluc reverse**	ACCCAGGAATCTCAGGAATGTCG
**Actin forward**	CATGTACGTTGCTATCCAGGC
**Actin reverse**	CTCCTTAATGTCACGCACGAT

Supplementary Note 1 Sequence information

The amino acid sequence (1-688) of luminGAVPO

MVFTLEDFVGDWRQTAGYNLDQVLEQGGVSSLFQNLGVSVTPIQRIVLSGENGLKIDIHVIIPYEGLSGDQMG

QIEKIFKVVYPVDDHHFKVILHYGTLVIDGVTPNMIDYFGRPYEGIAVFDGKKITVTGTLWNGNKIIDERLINPDG

SLLFRVTINGVTGWRLCERILASGGSGGSGMKLLSSIEQACDICRLKKLKCSKEKPKCAKCLKNNWECRYSPKTKR

SPLTRAHLTEVESRLERLERSIATRSHTLYAPGGYDIMGYLIQIMKRPNPQVELGPVDTSVALILCDLKQKDTPIVYA

SEAFLYMTGYSNAEVLGRNCRFLQSPDGMVKPKSTRKYVDSNTINTMRKAIDRNAEVQVEVVNFKKNGQRFV

NFLTMIPVRDETGEYRYSMGFQCETELQYPYDVPDYAEFQYLPDTDDRHRIEEKRKRTYETFKSIMKKSPFSGPT

DPRPPPRRIAVPSRSSASVPKPAPQPYPFTSSLSTINYDEFPTMVFPSGQISQASALAPAPPQVLPQAPAPAPAPA

MVSALAQAPAPVPVLAPGPPQAVAPPAPKPTQAGEGTLSEALLQLQFDDEDLGALLGNSTDPAVFTDLASVDN

SEFQQLLNQGIPVAPHTTEPMLMEYPEAITRLVTGAQRPPDPAPAPLGAPGLPNGLLSGDEDFSSIADMDFSAL

LSQISSDYKDDDDK*

The DNA sequence of 5xUAS$_G$-Gluc-P2A-insulin

CGGAGTACTGTCCTCCGAGCGGAGTACTGTCCTCCGACTCGAGCGGAGTACTGTCCTCCGATCGGAGTACTGTCC

TCCGCGAATTCCGGAGTACTGTCCTCCGAAGACGCTAGCGGGGGGCTATAAAAGGGGGTGGGGGCGTTCGTCCT

CACTCTAGATCTGCGATCTAAGTAAGCTTGGCATTCCGGTACTGTTGGTAAAGCCGGACGTCCTCTAGCCACCGCC

ACCAGCGCTGCCACCATGGGAGTCAAAGTTCTGTTTGCCCTGATCTGCATCGCTGTGGCCGAGGCCAAGCCCACC

GAGAACAACGAAGACTTCAACATCGTGGCCGTGGCCAGCAACTTCGCGACCACGGATCTCGATGCTGACCGCGG

GAAGTTGCCCGGCAAGAAGCTGCCGCTGGAGGTGCTCAAAGAGATGGAAGCCAATGCCCGGAAAGCTGGCTGC

ACCAGGGGCTGTCTGATCTGCCTGTCCCACATCAAGTGCACGCCCAAGATGAAGAAGTTCATCCCAGGACGCTGC

CACACCTACGAAGGCGACAAAGAGTCCGCACAGGGCGGCATAGGCGAGGCGATCGTCGACATTCCTGAGATTCC

TGGGTTCAAGGACTTGGAGCCCATGGAGCAGTTCATCGCACAGGTCGATCTGTGTGTGGACTGCACAACTGGCT

GCCTCAAAGGGCTTGCCAACGTGCAGTGTTCTGACCTGCTCAAGAAGTGGCTGCCGCAACGCTGTGCGACCTTT

GCCAGCAAGATCCAGGGCCAGGTGGACAAGATCAAGGGGGCCGGTGGTGACGGATCCGTGTACAAGGGAGCA

ACCAACTTTTCCCTGCTGAAGCAGGCAGGCGACGTGGAGGAGAATCCTGGACCCATGGCCCTGTGGATGCGCTT

CCTGCCCCTGCTGGCCCTGCTCGTCCTCTGGGAGCCCAAGCCTGCCCAGGCTTTTGTCAAACAGCACCTTTGTGG

TCCTCACCTGGTGGAGGCTCTGTACCTGGTGTGTGGGGAACGTGGTTTCTTCTACACACCCAAGTCCCGTCGTAA

AAGGGAGGACCCGCAAGTGCCACAACTGGAGCTGGGTGGAGGCCCGGAGGCCGGGGATCTTCAGACCTTGGC

ACTGGAGGTTGCCCGGCAGAAGCGTGGCATTGTGGATCAGTGCTGCACCAGCATCTGCTCCCTCTACCAACTGGA

GAACTACTGCAACTGA

Supplementary Note 2 The original WB figures of luminGAVPO

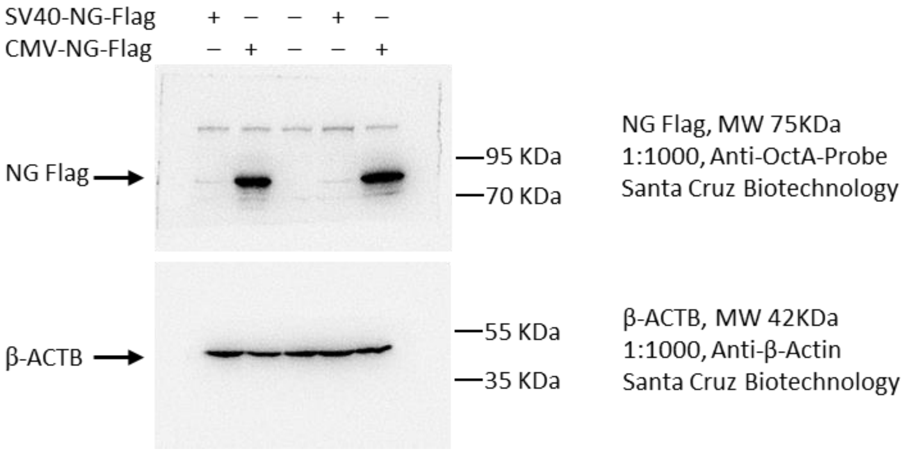

Supplement: Supplementary file 1 — Supplementary Information [file 41467_2021_20913_MOESM1_ESM.pdf]
